# Sexually transmitted infections and use of contraceptives in women living with HIV in Denmark – the SHADE cohort

**DOI:** 10.1186/s12879-016-1412-7

**Published:** 2016-02-16

**Authors:** Kristina Thorsteinsson, Steen Ladelund, Merete Storgaard, Frederikke Falkencrone Rønsholt, Isik Somuncu Johansen, Gitte Pedersen, Lars Nørregård Nielsen, Jesper Bonde, Henrik Westh, Niels Obel, Terese L. Katzenstein, Anne-Mette Lebech

**Affiliations:** Department of Infectious Diseases, Hvidovre, Copenhagen University Hospital, Copenhagen, Denmark; Clinical Research Center, Hvidovre, Copenhagen University Hospital, Hvidovre, Denmark; Department of Infectious Diseases, Skejby, Aarhus University Hospital, Aarhus, Denmark; Department of Infectious Diseases, Copenhagen University Hospital, Rigshospitalet, Copenhagen, Denmark; Department of Infectious Diseases, Odense University Hospital, Odense, Denmark; Department of Infectious Diseases, Aalborg University Hospital, Aalborg, Denmark; Department of Infectious Diseases, Hillerød Hospital, Hillerød, Denmark; Department of Pathology, Hvidovre, Copenhagen University Hospital, Copenhagen, Denmark; Institute of Clinical Medicine, University of Copenhagen, Copenhagen, Denmark; Department of Clinical Microbiology, Hvidovre, Copenhagen University Hospital, Copenhagen, Denmark

**Keywords:** Women living with HIV, Sexually transmitted infections, Screening, Contraceptive use, condom use, Hormonal contraception, Sexual activity

## Abstract

**Background:**

No Danish guidelines for screening of sexually transmitted infections (STIs) in women living with HIV (WLWH) exist, except for annual syphilis testing. Drug-drug interaction between hormonal contraceptives and some types of highly active antiretroviral therapy (HAART) occurs. We assessed prevalence of STIs, contraceptive choices and predictors of condom use in a cohort of WLWH in Denmark.

**Methods:**

WLWH consecutively enrolled during their outpatient visits from 2011 to 2012. Gynaecological examination and an interview were performed at entry and 6-month follow-up. Inclusion criteria were HIV-1 infection and ≥ 18 years of age. Exclusion criteria were pregnancy, alcohol- or drug abuse impeding adherence to the protocol. At entry, participants were tested (and where appropriate, treated according to guidelines) for *Chlamydia trachomatis*, *Neisseria gonorrhoeae*, syphilis, and *herpes simplex* (HSV-1 and HSV-2). At follow-up, predictors of condom use were estimated in sexually active WLWH.

**Results:**

In total, 334 of the 1,392 eligible WLWH in Denmark were included (median age and HIV duration: 42.5 and 11.3 years). *Chlamydia trachomatis* was present in four individuals (1 %), and six (2 %) tested positive for HSV-2 by PCR. None were positive for *Neisseria gonorrhoeae*, HSV-1 or had active syphilis. At follow-up, 252 (76 %) participated; 168 (70 %) were sexually active. Contraceptives were used by 124 (75 %); condoms were preferred (62 %). Having an HIV-negative partner predicted condom use (adjusted OR 3.89 (95 %CI 1.49-10.11)). In the group of participants of reproductive age without pregnancy desires 13 % used no birth-control. Possible drug-drug interaction between hormonal contraceptives and HAART was found in 13/14 WLWH receiving both kinds of medication.

**Conclusion:**

The prevalence of STIs in WLWH in Denmark was low. The need for annual STI screening is questionable. Condoms were preferred contraceptives, especially in WLWH with an HIV-negative partner. In this cohort, 13 % of WLWH of reproductive age were at risk of unintended pregnancies due to lack of birth-control. Finally, in the subgroup of WLWH receiving both hormonal contraceptives and HAART possible drug-drug interactions could occur.

**Electronic supplementary material:**

The online version of this article (doi:10.1186/s12879-016-1412-7) contains supplementary material, which is available to authorized users.

## Background

New attitudes towards condomless sex in people living with HIV-1 infection (PLHIV) have emerged as a result of among others the HPTN 052 trial that found reduced rates of sexual transmission of HIV in patients with early initiation of highly active antiretroviral therapy (HAART) [1] and the interim analysis of the PARTNER study reporting zero linked HIV transmissions from condomless sex within mixed-HIV-status couples, when the partner living with HIV had a viral load below 200 copies/mL [2]. Accordingly, sexual HIV transmission is thought to be negligible in patients adherent to HAART, with a suppressed viral load, and without sexually transmitted infections (STIs) [1, 2].

Increased incidences of STIs – serving as a proxy of condomless sex, especially among men who have sex with men (MSM) [3, 4] – are a risk to health and facilitate acquisition and transmission of HIV [3, 5]. Yet, data regarding the prevalence of STIs among women living with HIV (WLWH) are scarce [4, 5], and most existing studies focus on women newly diagnosed with HIV [5]. Guidelines from the European Aids Clinical Society (EACS) recommend that screening for STIs should be offered to all sexually active PLHIV at time of HIV diagnosis, annually thereafter or at any time STI symptoms are reported [6]. In Denmark, however, no guidelines for routine STI screening in PLHIV exist, except for annual syphilis testing [3]. Whether STI screening is needed in WLWH is unknown.

The trend towards condemless sex yields for alternate means of contraception. Most WLWH are of childbearing potential (10;11), which makes counselling on family planning and use of contraceptives a most relevant issue.

This article reports the first data from the Study on HIV, cervical Abnormalities and infections in women in Denmark (SHADE). Several gaps in evidence in caring for WLWH have been identified such as sexual health, gynaecological diseases and contraceptive use [7, 8]. The aim of the present study was to evaluate the prevalence of STIs in a cohort of WLWH and to assess whether routine screening for STIs in WLWH in Denmark is needed. Moreover, we describe sexual activity, contraceptive choices, and predictors of condom use in a cohort of WLWH in Denmark with free access to healthcare and HAART.

## Methods

### Setting

Denmark has a population of 5.6 million [9] and an estimated HIV prevalence among adults of 0.1 % [10]. Medical care, including HAART, is tax-paid and provided free-of-charge to all PLHIV. Treatment of HIV is restricted to eight specialized medical centres. Six of these centres (treating 97 % of Danish PLHIV) (Copenhagen University Hospital, Hvidovre, (HVH), Skejby, Aarhus University Hospital (AUH), Copenhagen University Hospital, Rigshospitalet (RH), Odense University Hospital (OUH), Aalborg University Hospital (AAUH), and Nordsjællands Hospital Hillerød (HIH)) participated in SHADE (see below).

### The SHADE cohort

The SHADE cohort is an ongoing, multicentre, prospective, observational cohort study of WLWH in Denmark attending regular outpatient care for their HIV infection. Study participants were consecutively enrolled during their outpatient visits from 1 February 2011 to 1 February 2012 and followed-up after 6 months. The study focuses on STIs, contraceptive choices, sexual activity, cervical abnormalities and cancer, human papillomavirus (HPV) infection, HIV disclosure and other aspects of living with HIV as a woman.

### Study population

WLWH in Denmark attending care in one of the Danish HIV centres. Inclusion criteria were HIV-1 infection and ≥ 18 years of age. Exclusion criteria were pregnancy, alcohol- or drug abuse impeding adherence to the protocol.

### Interview survey

Standardized interview questions were developed in consultation with clinical specialists and a statistician and appropriate revisions were made after a pilot study including ten HIV-negative volunteers. Questions were in Danish and closed-ended (Additional file 1 and Additional file 2; questionnaires in English). Double manual data entry of the questionnaires was performed, using the EpiData Entry program [11].

Trained doctors performed the standardized interviews regarding weight/height, tobacco and alcohol consumption, lifetime sexual partners, history of herpes and condyloma, symptoms from the lower abdomen, HPV vaccination status, adherence to HAART, and use of contraceptives.

At 6-month follow-up a similar interview regarding marital status, partner’s HIV status, sexual activity within the past 6 months, practice of anal sex, and possible reasons for no condom use was performed.

### Registries

#### The Civil Registration System (CRS)

The CRS is a national registry of all Danish residents [12]. A 10-digit personal identification number (PIN) is assigned to each individual at birth or immigration. The PIN was used as a linkage to the Danish HIV Cohort Study (DHCS).

#### Danish HIV Cohort Study (DHCS)

The DHCS is a prospective, observational, nationwide cohort study of all PLHIV seen at the Danish HIV clinics since 1January 1995 [13]. Data collection is ongoing, with continuous enrolment of newly diagnosed PLHIV. The database is updated annually and contains extensive data on demographics, date of HIV diagnosis, laboratory results etc.

### Microbiological testing

#### Chlamydia trachomatis (CT) and Neisseria gonorrhoeae (NG)

Samples were analysed according to local guidelines at the local departments of microbiology. At HVH, a BD ProbeTec ET Wet Swab was used for detection of *CT* and *NG* on oral, cervical, anal and first-void urine specimens (for the latter using the BD ProbeTec Urine Preservative Transport Kit (UPT)). At AAUH and RH, cervical and urethral specimens were examined for *CT* with a COBAS® Taqman® 48 analyzer (Roche Molecular Diagnostics, Indianapolis, IN, USA). Further, cervical, anal, urethral and oral specimens were obtained for culture of *NG* with charcoal swabs and transported in Stuart medium (SSI Diagnostica, Hillerød, Denmark). On specimens from AUH, OUH and HIH, the Gen-Probe Panther Aptima Combo2 test (San Diego, USA) was used for detection of *CT* and *NG*.

#### Syphilis

Until 1 July 2011 [14], syphilis serology was centralised at Statens Serum Institut (SSI) (the National Institute for Health Data and Diseases Control). Two non-treponemal tests were used; Wassermann’s reaction (WR) was done with a complement fixation technique, and rapid plasma reagin (RPR) was determined by agglutination [3]. Treponemal tests were performed on seroreactive samples [3]. After 1 July 2011 syphilis serology was decentralised to the local departments of microbiology performing a treponemal test detecting specific IgG and/or IgM antibodies to *Treponema* pallidum using the ADVIA Centaur® Syphilis assay (Siemens, US), the Syphilis TPA, from Vitros’ Immunodiagnostics, Ortho Clinical Diagnostics (High Wycombe, UK) or the Architect Syphilis TP (Abbott Japan, Japan: AST). If the antibody test was positive, non-treponemal tests (WR/RPR) were sent to SSI for confirmation. Patient files were examined regarding prior syphilis infection and treatment.

#### Herpes Simplex 1 and 2 (HSV-1 and HSV-2)

Diagnosis of HSV-1 and 2 was conducted at Department of Pathology, HVH. WLWH were swabbed once at entry and cervical samples were examined by the CLART Entherpex PCR (Genomica, Madrid, Spain). DNA extraction was done using the MagNAPure LC96 (Roche Molecular Systems, Rotkreuz, Ch). Samples showing an invalid outcome were retested, and the second result was considered definitive.

#### HIV RNA

Undetectable viral load was defined as a plasma HIV RNA load of <40 copies/mL, which was the highest level of sensitivity for testing in the observation period.

### Ethics, consent and permissions

At entry, written and oral informed consent was obtained from all participants. The study and the DHCS were approved by the Danish Data Protection Agency (2015–231–0126, 2012–58–0004 and 2012–41–0005). Further, the study was approved by the Danish Regional Committee on Health Research Ethics (approval numbers: H–3–2010–119 and H–2–2014–102).

### Statistical analyses

Continuous variables were summarized as median and interquartile ranges (IQR) and compared using Wilcoxon rank sum test. Categorical variables were reported as counts and percentages and compared by chi-square test or Fisher’s exact test as appropriate.

To calculate age and follow-up time for participants in the study and WLWH in the remaining DHCS 1 February 2012 (the last possible day of inclusion) was defined as day of inclusion.

Multivariate logistic regression analyses were performed to identify predictors of condom use in sexually active WLWH attending 6-month follow-up. Odds ratios (ORs) and 95 % confidence intervals (CI) were estimated and adjusted for seven candidate predictor variables chosen a priori, including age, race, HIV-RNA at inclusion, number of lifetime sexual partners, marital status, partner’s HIV status, and self-reported HSV infection prior to inclusion. To control for repeated testing, a combined *p*-value was estimated for variables spending more than one degree of freedom in the logistic regression.

Individuals with missing explanatory values were excluded from the multivariate regression analyses. The validity of the model was tested using the Hosmer and Lemeshow Goodness-of-Fit Test.

SAS statistical software version 9.3 (SAS Institute Inc., Cary, NC, USA) was used for data analysis and *p*-values <0.05 (two-sided) were considered statistically significant.

## Results

**Fig. 1:**
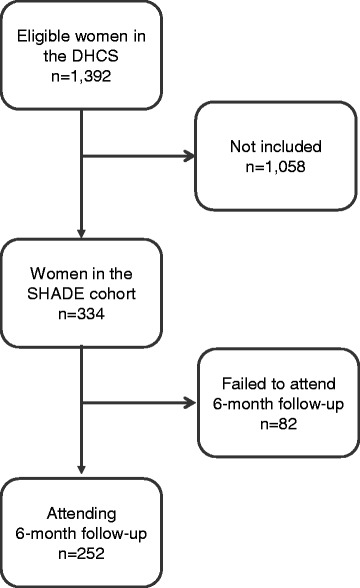
Flowchart of women in the Danish HIV Cohort Study (DHCS) and the SHADE cohort

Of the 1,392 WLWH alive and ≥18 years of age in the DHCS, 334 (24 %) consented to participate in the study (Fig. 1). At inclusion, median age was 42.5 years compared to 42.0 years (*p* = 0.22) in the remaining WLWH in the DHCS and HIV duration was 11.3 years compared to 10.6 years (*p* = 0.097) (Table 1). Other baseline characteristics are presented in Table 1.

**Table 1:** Baseline characteristics and prevalence of sexually transmitted infections in women living with HIV (WLWH) in the study and WLWH in the remaining Danish HIV Cohort Study (DHCS)

	WLWH in the study	WLWH in the remaining DHCS	*p*-value
Number of individuals	334 (24.0)	1,058 (76.0)	NA^1^
Follow-up (years), median (IQR)	11.3 (5.9–16.9)	10.6 (5.5–15.9)	0.097
Follow-up time, total (person-years)	3,853	11,183	NA^1^
Age at inclusion, median (IQR), (years)	42.5 (36.8–48.3)	42.0 (35.5–48.2)	0.22
Race, n (%)			
White	141 (42.6)	398 (39.5)	0.041^2^
Asian	44 (13.3)	114 (11.3)	
Black	143 (43.2)	461 (45.7)	
Other (missing)	3 (0.9)	35 (3.5)	
	(3)	(50)	
Place of HIV transmission, n (%)	114 (37.8)	326 (34.8)	0.11
Denmark	27 (8.9)	77 (8.2)	
Europe + US	128 (42.4)	416 (44.4)	
Africa	33 (10.9)	101 (10.8)	
Asia	0 (0)	17 (1.8)	
Other (missing)	(32)	(121)	
Mode of transmission, n (%)			
Heterosexual	294 (91.6)	799 (83.6)	0.0015
IDU	16 (5.0)	106 (11.1)	
Other (missing)	11 (3.4)	51 (5.3)	
	(13)	(102)	
CD4 count at inclusion (cells/μL), n (%)			
<200	12 (3.9)	79 (9.1)	0.012
200–350	51 (16.5)	138 (15.9)	
>350	247 (79.7)	650 (75.0)	
(missing)	(24)	(191)	
HAART at inclusion), n (%)			
Yes	317 (94.9)	866 (81.8)	<0.0001
No	17 (5.1)	192 (18.2)	
(missing)	(0)	(0)	
On HAART with HIV RNA < 40 copies/mL), n (%)			
Yes	250 (83.6)	576 (75.5)	0.0042
No	49 (16.4)	187 (24.5)	
(missing)	(18)	(103)	
Lifetime sexual partners, n (%)			
<5	99 (29.6)	-^3^	NA^1^
5–14	135 (40.4)		
15–25	45 (13.5)		
>25	53 (15.9)		
Does not wish to respond	2 (0.6)		
(missing)	(0)		
History of genital herpes infection, n (%)			
Yes	64 (19.2)	-^3^	NA^1^
No	227 (68.0)		
Unknown	43 (12.8)		
(missing)	(0)		
Symptoms from the lower abdomen, n (%)			
Yes	78 (23.3)	-^3^	NA^1^
No	237 (71.0)		
Does not wish to respond	19 (5.7)		
(missing)	(0)		
Outline of specific symptoms from the lower abdomen, n (% of women stating “yes” to lower abdominal symptoms)			
Vaginal discharge	30 (38.5)	-^3^	NA^1^
Burning sensation when urinating	8 (10.3)		
Abnormal menstrual bleeding	24 (30.8)		
Bleeding during sexual intercourse	6 (7.7)		
Pain while at rest	9 (11.5)		
Pain during sexual intercourse	15 (19.2)		
Other	34 (43.6)		
*Chlamydia trachomatis* infection, n (%)^4,5^	4 (1.2)	-^3^	NA^1^
Cervical	3		
Urethral	3		
*Neisseria gonorrhoeae* infection, n (%)^6^	0 (0)	-^3^	NA^1^
Cervical	0		
Urethral	0		
Rectal	0		
Pharyngeal	0		
Syphilis, n (%)^7^			
Any positive serologic test	8 (2.5)	-^3^	NA^1^
Positive serology requiring treatment	0 (0)		
HSV-1 infection, n (%)^8,9^	0 (0)	-^3^	NA^1^
HSV-2 infection, n (%)^8,9^	6 (1.9)	-^3^	NA^1^

^1^ NA = not applicable, ^2^ There was no difference in distribution of race between groups if the category “other” was removed from the “Race” variable (*p* = 0.45), ^3^ No information available, ^4^ 2/334 had no *Chlamydia trachomatis* sample*,* ^5^ Some patients were positive for Chlamydia in more than one anatomical location, ^6^ 1/334 had no *Neisseria gonorrhoeae* sample, ^7^ 9/334 had no syphilis test available, ^8^ HSV = *Herpes simplex virus,* ^9^ 16/334 had no sample for *herpes simplex virus type-1* or *-2*

### Sexually transmitted infections

At entry, four (1 %) women presented with *CT* (Table 1). Two of these reported condom use and two had lower abdominal symptoms. No participants tested positive for *NG* (Table 1). Positive serologic syphilis tests were found in eight (2 %) participants; all were interpreted as past infections; 7 (88 %) of these women were immigrants (Table 1). A history of genital herpes infection was stated by 64 (19 %). No participants were HSV-1 PCR positive, but six (2 %) tested HSV-2 positive by PCR; of whom all were on HAART (Table 1). The prevalence of STIs was too low to perform adjusted analyses aiming at predicting associated factors.

### Use of contraceptives – 6 month follow-up

A total of 252 (76 % of the SHADE cohort) women attended the 6 month follow-up (Fig. 1). Median time from inclusion to follow-up was median 6.2 (IQR 5.9–7.4) months. At follow-up, 168 (69 %) reported sexual activity in the past 6 months - of these 165 (65 %) responded to the question regarding contraceptive use (Table 2). Overall, 124 (75 %) of the sexual active women used contraceptives (Table 2). Among the 41 women using no contraceptives, 29 were of reproductive age (<50 years) and 10 of these stated a desire for pregnancy – leaving 19 (13 % of the sexually active WLWH in the reproductive age) without birth control.

**Table 2:** Characteristics on marital status, sexual activity and contraception at 6-month follow-up (*n* = 252)

	WLWH in the study
Marital status, n (%)	
Married	105 (43.0)
Cohabitating	35 (14.3)
Regular partner (not cohabitating)	27 (11.1)
Single	77 (31.6)
(missing)	(8)
Partner’s HIV status, n (%)	
HIV-positive	50 (29.1)
HIV-negative	118 (68.6)
Not tested/do not know	4 (2.3)
(missing)	(80)
Sexual activity in the past 6 months, n (%)	
Yes	168 (68.6)
No	77 (31.4)
(missing)	(7)
Current use of contraception in sexually active women, n (%)	
Condom	84 (50.9)
Condom + hormonal contraception	6 (3.6)
Condom + intra uterine device	1 (0.6)
Condom + sterilization	11 (6.7)
Hormonal contraception	9 (5.5)
Intra uterine device	5 (3.0)
Sterilization	6 (3.6)
Other	2 (1.2)
Nothing	41 (24.9)
(missing)	(3)
Reasons for no condom use in sexually active women, n (%)^1,2^	
Patient’s wish	2 (3.2)
Partner’s wish	11 (17.7)
Joint decision	25 (40.3)
Partner has HIV	23 (37.1)
Attempts to become pregnant	10 (16.1)
HIV RNA is low	13 (21.0)

^1^More than one answer allowed, n (%) does not add up to 100 %, ^2^N (% of women responding to the specific question)

Condom use alone or as part of dual protection were stated by 102 (62 %) participants (Table 2). Having an HIV-negative partner predicted condom use (OR 3.89 (95 % CI 1.49–10.11)) (Table 3). A sensitivity analysis of the adjusted analysis was performed to check for the effect of missing values on outcome by adding an extra category with missing values. This had no impact on the estimates.

**Table 3:** Unadjusted and adjusted odds ratios for predictors of condom use in sexually active women stating use of contraception at 6-month follow-up (*n* = 165)

Predictors of condom use	No condom use (*n* = 63)	Condom use (*n* = 102)	Unadjusted odds ratios	*p*-value	Mutually adjusted odds ratios^1^	*p*-value
Age at 1 February 2011 (inclusion), (years)						
>50	15 (57.7)	11 (42.3)	1.00	–	1.00	–
30–49	44 (34.9)	82 (65.1)	2.54 (1.08–6.00)	0.034	2.30 (0.66–7.99)	0.19
18–29	4 (30.8)	9 (69.2)	3.07 (0.75–12.59)	0.12	1.14 (0.11–11.54)	0.91
(missing)	(0)	(0)				
Combined *p*-value				0.089		0.36
Race, n (%)						
White	27 (38.6)	43 (61.4)	1.00	–	1.00	–
Asian	11 (45.8)	13 (54.2)	0.74 (0.29–1.89)	0.53	0.62 (0.19–2.07)	0.44
Black	24 (34.3)	46 (65.7)	1.20 (0.60–2.40)	0.60	0.66 (0.25–1.72)	0.39
(missing)	(1)	(0)				
Combined *p*-value				0.60		0.64
HIV RNA at inclusion, n (%)^2^						
Undetectable	45 (35.2)	83 (64.8)	1.00	–	1.00	–
Detectable	13 (54.2)	11 (45.8)	0.46 (0.19–1.11)	0.083	0.86 (0.30–2.49)	0.78
(missing)	(5)	(8)				
Combined *p*-value				0.083		0.78
Lifetime sexual partners at inclusion, n (%)						
<4	22 (47.8)	24 (52.2)	1.00	–	1.00	–
5–14	25 (36.8)	43 (63.2)	1.58 (0.74–3.37)	0.24	1.40 (0.52–3.79)	0.51
15–25	9 (34.6)	17 (65.4)	1.73 (0.64–4.68)	0.28	0.94 (0.23–3.80)	0.93
>25	7 (28.0)	18 (72.0)	2.36 (0.83–6.72)	0.11	0.96 (0.23–4.00)	0.96
(missing)	(0)	(0)				
Combined *p*-value				0.38		0.85
Marital status, n (%)						
Married	35 (41.2)	50 (58.8)	1.00	–	1.00	–
Cohabitating	17 (53.1)	15 (46.9)	0.62 (0.27–1.40)	0.25	0.55 (0.20–1.50)	0.24
Regular partner (not cohabitating)	8 (33.3)	16 (66.7)	1.40 (0.54–3.63)	0.49	1.01 (0.31–3.23)	0.99
Single	3 (12.5)	21 (87.5)	4.90 (1.36–17.70)	0.015	–	0.99
(missing)	(0)	(0)				
Combined *p*-value				0.030		0.67
Partner’s HIV status, n (%)						
HIV-positive	30 (68.2)	14 (31.8)	1.00	–	1.00	–
HIV-negative	27 (28.7)	67 (71.3)	5.32 (2.45–11.55)	<0.000	3.89 (1.49–10.11)	0.0054
Not tested/do not know	2 (50.0)	2 (50.0)	2.14 (0.27–16.81)	1	1.01 (0.05–19.87)	0.99
(missing)	(4)	(19)		0.47		
Combined *p*-value				0.0001		0.014
Selfreported HSV^3^ infection prior to inclusion, n (%)						
Yes	14 (43.8)	18 (56.2)	1.00	–	1.00	–
No	40 (36.0)	71 (64.0)	1.38 (0.62–3.07)	0.43	1.75 (0.66–4.63)	0.26
Do not know (missing)	9 (40.9)	13 (59.1)	1.12 (0.37–3.38)	0.84	1.63 (0.43–6.25)	0.47
Combined *p*-value				0.70		0.53

^1^The validity of the model was tested using the Hosmer and Lemeshow Goodness-of-Fit Test, ^2^HIV-RNA <40 copies/ml, ^3^HSV = Herpes simplex virus infection

Fifteen (9 %) of the sexually active women reported use of hormonal contraception (HC) alone or as part dual protection; of these; 14 were on HAART and 13 (93 %) received a regimen containing either efavirenz, nevirapine, etravirine, atazanavir/ritonavir or darunavir/ritonavir with possible drug-drug interactions with HC.

## Discussion

In this multicenter, prospective, observational cohort of WLWH in Denmark, we found a low prevalence of STIs thus questioning the need for annual STI screening in WLWH as recommended by e.g. the EACS guidelines [6]. The majority of sexually active WLWH used condoms; primarily when being in a discordant relationship. More than 10 % of sexually active WLWH in the reproductive age were at risk of unintended pregnancy. Finally, the majority of WLWH on both HAART and HC received a combination with potential drug-drug interactions that could decrease contraceptive efficacy.

WLWH in SHADE was no different from WLWH in the remaining nationwide DHCS with regards to age, HIV and race, however they were more likely to be sexually infected with HIV, having a higher CD4 count and a greater probability of being on HAART with a suppressed viral load. We attribute these differences to the exclusion criteria regarding drug abuse.

The viral load of HIV in plasma is a major determinant of HIV transmission [15] and the correlation between plasma and genital viral load is strong [1, 15]. Genital HIV shedding is also influenced by local factors such as genital ulcers, *CT, NG* and HSV [5, 15–17]. The increased incidences of STIs in Western cohorts of PLHIV [3, 4] is therefore of concern. Especially, asymptomatic STIs are worrying due to complications related to the STI in question and rise in HIV infectiousness [5]. In WLWH, however, data on STIs, contraceptive choices, and sexual health are scarce [4, 8].

### Sexually transmitted infections

Only four (1 %) participants presented with *CT,* two of whom had symptoms. No participants were diagnosed with *NG.* In comparison, the point prevalence of *CT* and *NG* in WLWH has been reported to be 1–27 % in studies carried out in the Western setting, with the highest prevalence found in cohorts of young, newly diagnosed WLWH [5, 18]. The long median HIV duration of 11 years in the present cohort impedes comparison of these studies. Interestingly, an older Danish study on the general population found a comparable prevalence of *CT* <2 % in women older than 32 years of age and <1 % in women older than 43 years of age [19].

The increasing rates of syphilis in MSM living with HIV suggest a sexual high-risk behaviour [3] that is not prevalent amongst WLWH in this study. Whereas more than 70 % of women reported <15 lifetime sexual partners, this number is markedly higher in MSM cohorts [20]. Also, compared to international female HIV cohorts the number is low; in the Women’s Interagency HIV Study (WIHS) 31 % had 10–49 lifetime sexual partners and 24 % reported >50 sexual partners [21]. The prevalence of syphilis in cohorts of WLWH in Western settings is 2–13 % - peaking amongst newly diagnosed WLWH [5]. In the present study no syphilis cases were seen, however, the majority of WLWH presenting with positive syphilis serology indicating a prior infection were immigrants, which suggests a lower threshold for screening in this subgroup.

Up to half of all newly diagnosed PLHIV demonstrate HSV-2 infection, which has been ascribed to the association between ulcerative STIs and HIV [22]. Here, more than 40 % of participants reported to be infected with HIV in Africa, where HIV and HSV co-infection is highly prevalent [23]. Yet, no women were tested positive by PCR of HSV-1 and only 2 % tested PCR positive for HSV-2, while about one fifth reported a history of genital herpes infection. All participants shedding HSV-2 were on HAART, which is in line with studies finding no attenuation of HSV-2 reactivation in individuals on suppressive HAART [24]. In studies of HSV-2 seropositive WLWH, a markedly higher prevalence of 11–23 % of HSV-2 shedding were found [24, 25].

### Use of contraceptives

Studies on contraceptive use in WLWH are strikingly scarce [8, 26]. The proportion of participants reporting use of any contraceptive method was higher in the study group, than the estimated proportion of contraceptive use in developed regions of the world and the global proportion in 2011; 75 %, 70 % and 63 %, respectively [27]. However, >10 % of sexually active participants were in the reproductive age, had no pregnancy desire, and nonetheless used no birth control.

Though, the number of lifetime sexual partners was low compared to the WIHS, the number of women reporting to be sexually active in the past 6 months was comparable at around 70 % [21]. Preferred contraceptives were condoms used by 62 % of sexually active women. In comparison, 26–80 % reported using a barrier contraceptive method at least intermittently in cohorts of young PLHIV [18, 28, 29]. Condom use in the present study was predicted by having an HIV-negative partner.

The metabolism of both HC and some non-nucleoside reverse-transcriptase inhibitors (NNRTIs) and protease inhibitors (PIs) is dependent on the cytochrome P450 system in the liver [7, 26]. The NNRTIs efavirenz and nevirapine both decrease effectiveness of HC [7], while no dose adjustment is needed for rilpivirine, etravirine can be co-administered with 35 μg estrogen [7]. Prescription of PIs boosted with ritonavir is not recommended in combination with HC with the exception of atazanavir, which however requires a dose of at least 30 μg estrogen [7]. In the present study, 13 out of 14 participants on both HAART and HC were on a regimen with possible drug-drug interactions and therefore at risk of reduced contraceptive efficacy.

The strengths of this study include the prospective nature and the use of validated laboratory tests. Additionally, the standardized methods of data collection and the performance of double manual data entry enhanced accuracy of data input and integrity of the interview results. Lastly, the ability to link to the nationwide, complete DHCS with basic data on all PLHIV in Denmark optimises results.

Limitations of the study were that women were only recruited on days where nurses or doctors responsible of the study were present in the outpatient clinic. However, due to the DHCS differences between groups can be addressed. Though, we present a low point prevalence of STIs, incidence of STIs in this cohort has not been examined. Finally, self-reported data especially on sexual history may be unreliable [30].

## Conclusion

Overall, the prevalence of STIs in WLWH in Denmark was low. Screening for STIs at entry of HIV care may prove valuable as shown by other cohorts [5] and due to the fact that especially immigrants presented with positive syphilis serology indicating prior infection. However, annual screening for STIs in WLWH attending routine HIV care is of questionable value. Three quarters of sexually active WLWH used contraception. Condoms were preferred contraceptives, especially when having an HIV-negative partner. The majority of WLWH receiving both HAART and HC were on a regimen with possible drug-drug interactions with ensuing risk of decreased contraceptive efficacy. The discrepancy between the low rates of STIs and the rather low proportion of condom use can probably be explained by the relatively low number of lifetime sexual partners in this cohort. In our opinion, there is an unmet need for contraceptive counselling in WLWH.

## Additional files

Additional file 1:
**Questionnaire 1.** (DOC 126 kb) Additional file 2:
**Questionnaire 2.** (DOC 123 kb)

## Abbreviations

CI: confidence interval
CT: chlamydia trachomatis
CRS: Civil Registration System
DHCS: Danish HIV Cohort Study
HAART: highly active antiretroviral therapy
HC: hormonal contraception
HPV: human papillomavirus
HSV: Herpes simplex virus
MSM: men who have sex with men
NG: Neisseria gonorrhoeae
NNRTI: non-nucleoside reverse-transcriptase inhibitor
NRTI: nucleoside reverse-transcriptase inhibitor
OR: odds ratio
PI: protease inhibitor
PIN: personal identification number
PLHIV: people living with HIV
SHADE: Study on HIV, cervical Abnormalities and infections in women in Denmark
SSI: Statens Serum Institut (the National Institute for Health Data and Diseases Control)
STI: sexually transmitted infections
WLWH: women living with HIV
WIHS: Women’s Interagency HIV Study
