# Supplementary material for: Sexually transmitted infections and use of contraceptives in women living with HIV in Denmark – the SHADE cohort
Source: BMC Infect Dis. 2016 Feb 16;16:81. doi: 10.1186/s12879-016-1412-7 (PMC4754814; doi:10.1186/s12879-016-1412-7)
Supplement: Additional file 1: — Questionnaire 1. (DOC 126 kb) [file 12879_2016_1412_MOESM1_ESM.doc]

**Interview at visit 1 (inclusion):**

| **Patient name**  **PIN**  **(Label)** |  | Patient number | □□□□ |
| --- | --- | --- | --- |

| **Phone number:** (if patient accepts contact per phone) | □□ □□ □□ □□ |
| --- | --- |
| **E-mail:** (if patient accepts contact per mail) |  |

| **Date of inclusion** (dd.mm.yyyy): | □□ □□ □□□□ |
| --- | --- |

| **A**  **1** | **Inclusion criteria** | | | |
| --- | --- | --- | --- | --- |
|  | **I** | Age ≥ 18 years | □  Yes | ■  No |
|  | **ii** | Capable of giving informed consent | □  Yes | ■  No |

| **B**  **1** | **Exclusion criteria** | | | |
| --- | --- | --- | --- | --- |
|  | **i** | Alcohol and/or drug abuse impeding adherence to protocol | ■  Yes | □  No |
|  | **ii** | Pregnancy | ■  Yes | □  No |
|  | **iii** | Hysterectomy (unless the cervix is preserved) | ■  Yes | □  No |

| C1 | **Patient weight/height** | |
| --- | --- | --- |
|  | Weight (kg) | □□□ kg |
|  | Height (cm) | □□□ cm |

| **D**  **1** | Tobacco use | | | | | |
| --- | --- | --- | --- | --- | --- | --- |
|  | **i** | Current smoker (if no, go to **E.1**) | □ Yes (1) | □  Former  (2) | | □ No  (3) |
|  | **ii** | Cigarettes | □  Yes (1) | | □  No (2) | |
|  |  | Cigarettes per day | □□□ number | | | |
|  |  | Number of years the patient has smoked cigarettes | □□ years | | | |
|  |  | Cigarillos | □  Yes (1) | | □  No (2) | |
|  |  | Cigarillos per day | □□ number | | | |
|  |  | Number of years the patient has smoked cigarillos | □□ years | | | |
|  |  | Cigars | □  Yes (1) | | □  No (2) | |
|  |  | Cigars per day | □□ number | | | |
|  |  | Number of years the patient has smoked cigars | □□ years | | | |
|  |  | Pipe | □  Yes (1) | | □  No (2) | |
|  |  | Pipe bowls per day | □□ number | | | |
|  |  | Number of years the patient has smoked pipe | □□ years | | | |

| **E**  **1** | Alcohol consumption | |
| --- | --- | --- |
|  | Alcohol units per week | □□□ number |

| F1 | **Contraceptive use** (please place one cross only) | |
| --- | --- | --- |
|  | Nothing | □(0) |
|  | Condom | □(1) |
|  | Hormonal contraceptives (oral contraceptives/birth control implant) | □(2) |
|  | IUD | □(3) |
|  | Sterilization | □(4) |
|  | Condom + hormonal contraceptives | □(5) |
|  | Condom + IUD | □(6) |
|  | Condom + sterilization | □(7) |
|  | Other | □(8) |
|  | Does not wish to respond | □(9) |

| **G1** | Sexual debut | | |
| --- | --- | --- | --- |
|  | i | Yes | □(1) |
|  |  | No (go to **I.1**) | □(2) |
|  |  | Does not wish to respond (go to **H.1**) | □(3) |
|  | ii | Age at sexual debut (00 = does not know) | □□ |

| **H1** | **Number of lifetime sexual partners** (please place one cross only) | |
| --- | --- | --- |
|  | <4 | □(1) |
|  | 5-9 | □(2) |
|  | 10-14 | □(3) |
|  | 15-25 | □(4) |
|  | 26-40 | □(5) |
|  | >40 | □(6) |
|  | Does not wish to respond | □(7) |

| I1 | **History of condyloma?** (please place one cross only) | |
| --- | --- | --- |
|  | Yes | □(1) |
|  | No | □(2) |
|  | Does not know | □(3) |

| J1 | **History of genital herpes?** (please place one cross only) | |
| --- | --- | --- |
|  | Yes | □(1) |
|  | No | □(2) |
|  | Does not know | □(3) |

| K1 | **Symptoms from the lower abdomen?** (please place one cross only) | |
| --- | --- | --- |
|  | Yes | □(1) |
|  | No (go to **M.1**) | □(2) |
|  | Does not wish to respond (go to **M.1**) | □(3) |

| L1 | **Symptoms from the lower abdomen?** (you may place several crosses) | | |
| --- | --- | --- | --- |
|  | Vaginal discharge | □  Yes (1) | □  No (2) |
|  | Burning sensation when urinating | □  Yes (1) | □  No (2) |
|  | Abnormal menstrual bleeding | □  Yes (1) | □  No (2) |
|  | Bleeding during sexual intercourse | □  Yes (1) | □  No (2) |
|  | Pain while at rest | □  Yes (1) | □  No (2) |
|  | Pain during sexual intercourse | □  Yes (1) | □  No (2) |
|  | Other | □  Yes (1) | □  No (2) |

| M1 | **HPV vaccination** (please place one cross only) | |
| --- | --- | --- |
|  | No (go to **Q.1**) | □(0) |
|  | Yes, Gardasil | □(1) |
|  | Yes, Cervarix | □(2) |
|  | Yes, does not know the name of the vaccine | □(3) |

| N1 | **Year of first HPV vaccination** | |
| --- | --- | --- |
|  | Year of first vaccination (yyyy) (0000 = does not know) | □□□□ |

| O1 | **Reason for HPV vaccination** (please place one cross only) | |
| --- | --- | --- |
|  | Patient’s own initiative | □(1) |
|  | Doctors recommendation due to condyloma | □(2) |
|  | Doctors recommendation due to other reasons | □(3) |
|  | Does not know | □(4) |

| P1 | **Number of vaccinations** | |
| --- | --- | --- |
|  | Does not know | □(0) |
|  | 1 | □(1) |
|  | 2 | □(2) |
|  | 3 | □(3) |

| Q1 | **Adherence to HIV medicine – number of forgotten doses of ART within the past 30 days**  (please place one cross only) | |
| --- | --- | --- |
|  | Does not receive antiretroviral therapy | □(0) |
|  | 0 | □(1) |
|  | 1-4 | □(2) |
|  | 5-9 | □(3) |
|  | 10-15 | □(4) |
|  | >15 | □(5) |
