# Supplementary material for: Sexually transmitted infections and use of contraceptives in women living with HIV in Denmark – the SHADE cohort
Source: BMC Infect Dis. 2016 Feb 16;16:81. doi: 10.1186/s12879-016-1412-7 (PMC4754814; doi:10.1186/s12879-016-1412-7)
Supplement: Additional file 2: — Questionnaire 2. (DOC 123 kb) [file 12879_2016_1412_MOESM2_ESM.doc]

**Interview at visit 2 (6 month):**

| **Patient name**  **PIN**  **(Label)** |  | Patient number | □□ □□ |
| --- | --- | --- | --- |

| **Date of 2nd visit** (dd.mm.yyyy): | □□ □□ □□□□ |
| --- | --- |

| **Phone number** (if patient accepts this) | □□ □□ □□ □□ |
| --- | --- |
| **E-mail** (if patient accepts this) |  |

| A2 | **Marital status?** (please place one cross only) | |
| --- | --- | --- |
|  | Married | □(1) |
|  | Cohabitating | □(2) |
|  | Regular partner (not cohabitating) | □(3) |
|  | Single | □(4) |
|  | Does not wish to respond (go to **C.2**) | □(5) |

| B2 | **Partner’s HIV status?** (please place one cross only) | |
| --- | --- | --- |
|  | HIV-positive | □(1) |
|  | HIV-negative | □(2) |
|  | Not tested | □(3) |
|  | Does not know | □(4) |
|  | Does not wish to respond | □(5) |

| C2 | **Sexual activity within the past 6 months?** (please place one cross only) | |
| --- | --- | --- |
|  | Yes | □(1) |
|  | No (go to **F.2**) | □(2) |
|  | Does not wish to respond (go to **F.2**) | □(3) |

| D2 | **If yes, when was the last time you had sex?** (please place one cross only) | |
| --- | --- | --- |
|  | Within the past week | □(1) |
|  | Within the past month | □(2) |
|  | Within the past 6 months | □(3) |
|  | Does not wish to respond | □(5) |

| **E2** | **Number of sexual partners since last visit** (please place one cross only) | |
| --- | --- | --- |
|  | 0 | □(0) |
|  | 1 | □(1) |
|  | 2-4 | □(2) |
|  | 5-9 | □(3) |
|  | 10-14 | □(4) |
|  | 15-25 | □(5) |
|  | 26-40 | □(6) |
|  | >40 | □(7) |
|  | Does not wish to respond | □(8) |

| F2 | **Ever practiced anal sex?** (please place one cross only) | |
| --- | --- | --- |
|  | Yes | □(1) |
|  | No | □(2) |
|  | Does not wish to respond | □(3) |

| G2 | **Symptoms from the lower abdomen?** (please place one cross only) | |
| --- | --- | --- |
|  | Yes (go to **H.2**) | □(1) |
|  | No (go to **I.2**) | □(2) |
|  | Does not wish to respond (go to **I.2**) | □(3) |

| H2 | **Outline of specific symptoms from the lower abdomen?** (you may place several crosses) | | |
| --- | --- | --- | --- |
|  | Vaginal discharge | □  Yes (1) | □  No (2) |
|  | Burning sensation when urinating | □  Yes (1) | □  No (2) |
|  | Abnormal menstrual bleeding | □  Yes (1) | □  No (2) |
|  | Bleeding during sexual intercourse | □  Yes (1) | □  No (2) |
|  | Pain while at rest | □  Yes (1) | □  No (2) |
|  | Pain during sexual intercourse | □  Yes (1) | □  No (2) |
|  | Other | □  Yes (1) | □  No (2) |

| **I**  **2** | **HPV vaccination since last visit?** (please place one cross only) | |
| --- | --- | --- |
|  | No (go to **M.2**) | □(0) |
|  | Yes, Gardasil | □(1) |
|  | Yes, Cervarix | □(2) |
|  | Yes, do not know name of vaccine | □(3) |

| J2 | **Date of first HPV vaccination?** | |
| --- | --- | --- |
|  | Month and year (mm.yyyy) (00.0000 = does not know) | □□ □□□□ |

| K2 | **Reason for HPV vaccination?** (please place one cross only) | |
| --- | --- | --- |
|  | Patient’s own initiative | □(1) |
|  | Doctors recommendation due to condyloma | □(2) |
|  | Doctors recommendation due to other reasons | □(3) |
|  | As part of a HPV vaccination trial | □(4) |
|  | As part of the Danish vaccination program (women below 26 years of age) | □(5) |
|  | Does not know | □(6) |

| L2 | **Number of HPV vaccinations?** | |
| --- | --- | --- |
|  | Does not know | □(0) |
|  | 1 | □(1) |
|  | 2 | □(2) |
|  | 3 | □(3) |

| **M**  **2** | **Current use of contraceptives?** (please place one cross only) | |
| --- | --- | --- |
|  | Nothing (go to **P.2**) | □(0) |
|  | Hormonal contraceptives (oral contraceptives/birth control implant)  (go to **P.2**) | □(1) |
|  | IUD (go to **P.2**) | □(2) |
|  | Sterilization (if yes, go to **N.2**) | □(3) |
|  | Condom  (End of questionnaire) | □(4) |
|  | Condom + oral contraceptives/birth control implant  ( End of questionnaire) | □(5) |
|  | Condom + IUD  ( End of questionnaire ) | □(6) |
|  | Condom + sterilization (if yes, go to **N.2**) | □(7) |
|  | Other (go to **P.2**) | □(8) |
|  | Does not wish to respond  (End of questionnaire) | □(9) |

| N **2** | **If the patient is sterilized: Where you sterilized after your HIV diagnosis?**  (please place one cross only) | |
| --- | --- | --- |
|  | Yes | □(1) |
|  | No | □(2) |
|  | Does not wish to respond | □(3) |

| O2 | **Have you subsequently regretted being sterilized?** (please place one cross only) | |
| --- | --- | --- |
|  | Yes | □(1) |
|  | No | □(2) |
|  | No, the patient already had the desired number of children | □(3) |
|  | Does not wish to respond | □(4) |

| P2 | **If the patient does not use condom: What are the reasons for no condom use?**  (you may place several crosses) | | |
| --- | --- | --- | --- |
|  | Patient’s wish | □  Yes (1) | □  No (2) |
|  | Partner’s wish | □  Yes (1) | □  No (2) |
|  | Joint decision | □  Yes (1) | □  No (2) |
|  | Partner has HIV | □  Yes (1) | □  No (2) |
|  | Attempts to become pregnant | □  Yes (1) | □  No (2) |
|  | HIV RNA is low | □  Yes (1) | □  No (2) |
|  | The patient is not sexually active | □  Yes (1) | □  No (2) |
|  | Does not wish to respond | □  Yes (1) | □  No (2) |
